# Application of NaCl-supplemented antimicrobial susceptibility testing conditions to marine bacterial isolates

**DOI:** 10.71150/jm.2606005

**Published:** 2026-09-30

**Authors:** Daegyu Lee, Jin Lee, Hye Won Hong, Tatsuya Unno, Dukki Han

**Affiliations:** 1 Department of Marine Convergence Science, Kangwon National University, Gangneung 25457, Republic of Korea; 2 ShinSung Bio Laboratory of ShinSung Biopharm Inc, Gangneung 64122, Republic of Korea; 3 Forensic DNA Division, National Forensic Service, Wonju 26460, Republic of Korea; 4 Department of Biological Sciences and Biotechnology, Chungbuk National University, Cheongju 28644, Republic of Korea; 5 EcoTrace, Gangneung 25457, Republic of Korea

**Keywords:** antimicrobial resistance, MIC, NaCl, marine bacteria, *Vibrio*

## Abstract

Antimicrobial resistance (AMR) in marine environments remains difficult to assess under standard antimicrobial susceptibility testing conditions, which may not adequately reflect the physiological requirements of marine-derived bacteria. In this study, we applied NaCl-supplemented susceptibility testing conditions to bacterial isolates recovered from coastal environments and marine organism guts to evaluate their practical applicability and limitations. A total of 927 isolates were obtained from lagoon, seawater, soil, and gut samples of abalone, crab, eel, halibut, and salmon. Of these, 365 isolates grew on standard Mueller–Hinton (MH) medium, whereas 562 isolates were not evaluable under standard MH conditions. Taxonomic analysis showed that the isolate collection was dominated by *Proteobacteria* and *Firmicutes*, with *Vibrio* as the most abundant genus. Disk diffusion assays of the 365 MH-grown isolates revealed differences in screening-level reduced susceptibility profiles among sample groups, and putative multidrug resistance was frequently observed in *Vibrio* isolates from abalone and seawater. For isolates not evaluable under standard MH conditions, minimum inhibitory concentration (MIC) analysis was performed using 2% NaCl-supplemented MH broth. Under these conditions, 115 isolates showed stable growth and distinct antibiotic-dependent MIC profiles. Chloramphenicol and tetracycline inhibited most isolates at relatively low concentrations, whereas reduced susceptibility to penicillin was relatively high in isolates from seawater, abalone, and eel. Overall, these findings suggest that standard MH conditions alone may be insufficient for evaluating antibiotic responses in marine- and brackish-origin bacteria, and that 2% NaCl-supplemented MH broth can serve as a practical supplementary condition for selected marine-derived isolates.

## Introduction

From a One Health perspective, antimicrobial resistance (AMR) is a cross-sectoral issue linking humans, animals, and the environment. Aquatic ecosystems are particularly important for understanding environmental AMR because they serve as major reservoirs where antibiotics, resistant bacteria, and resistance genes can accumulate and spread, and where genetic exchange can occur among microorganisms (Lajqi Berisha et al., 2024; Marti et al., 2014; Montiel-Mora et al., 2026; Thornber et al., 2020). Although numerous studies have reported AMR monitoring results in aquatic ecosystems, most have focused primarily on freshwater environments (Montiel-Mora et al., 2026). In contrast, several studies have addressed AMR in marine environments and have also highlighted the difficulties in risk assessment and the limitations of resistance monitoring in these systems (Lu et al., 2018; Zheng et al., 2021). However, antimicrobial susceptibility and resistance in microorganisms from marine environments and marine organisms have not been sufficiently evaluated under variable salinity conditions.

Current antimicrobial susceptibility testing is primarily based on Clinical and Laboratory Standards Institute (CLSI) guidelines, particularly disk diffusion and broth dilution methods using Mueller–Hinton (MH) media, which are useful for standardizing procedures and comparing results among clinical bacterial isolates (Humphries et al., 2021). However, these standard conditions may not adequately reflect the actual growth characteristics and antibiotic responses of marine-derived microorganisms or bacteria that are sensitive to salinity (Ruangpan and Tendencia, 2004). Many marine-derived microorganisms require higher salt concentrations than those provided in conventional clinical media; accordingly, Marine Agar (MA) or Mueller–Hinton Agar (MHA) supplemented with NaCl have been used (Ruangpan and Tendencia, 2004). Nevertheless, variations in salinity can influence both the physicochemical behavior of antibiotics and the physiological responses of bacteria, potentially causing phenotypic interference in susceptibility test results (Li et al., 2025). Therefore, it is important to establish testing conditions that reflect the physiological requirements of marine-derived microorganisms while maintaining comparability with standard susceptibility testing methods.

To evaluate antimicrobial resistance in microorganisms, it is necessary to quantitatively determine how changes in NaCl concentration affect susceptibility test results (Ma et al., 2021). A recent study evaluated antimicrobial susceptibility testing conditions suitable for marine environments using standard reference strains, and comparisons between MH and MA media, together with NaCl gradient experiments, showed that the effects of NaCl varied depending on the antibiotic and the strain (Han, 2026). These findings demonstrated the need to optimize testing conditions for marine-derived microorganisms and suggested the potential for an improved testing approach. However, results obtained only from standard reference strains are not sufficient to determine whether the same conditions can be reliably applied to diverse microbial communities isolated from marine environments. Marine microorganisms exhibit much broader physiological diversity than standard strains, and their salt tolerance and growth characteristics may vary greatly depending on their habitat. Therefore, further studies are needed to determine whether antimicrobial susceptibility testing conditions improved using standard reference strains can be reproducibly applied to marine isolates, and to identify which antibiotics require particularly careful interpretation.

In this study, we applied previously proposed NaCl-supplemented antimicrobial susceptibility testing conditions (Han, 2026) to diverse marine bacterial isolates and evaluated their practical applicability and limitations, stability, reproducibility, and interpretability. To this end, basal growth assessment, disk diffusion, and minimum inhibitory concentration (MIC)-based susceptibility testing were performed using isolates obtained from different environments, and the effects of NaCl conditions were compared according to antibiotic type, taxonomic characteristics, and habitat. This approach extends preliminary evaluation beyond standard reference strains to environmental isolates and may provide baseline information for future refinement of antimicrobial susceptibility testing conditions for marine-derived microorganisms.

## Materials and Methods

### Sample collection and bacterial isolation

In this study, bacterial isolates were obtained from environmental samples, including lagoon water, seawater, and soil, and from gut samples of marine organisms, including abalone, crab, eel, halibut, and salmon, with five to twelve isolates collected from each individual sample. Detailed sample information is provided in Table 1. Seawater-derived isolates were obtained from coastal seawater collected around Ulleung Island, Republic of Korea. Soil-derived isolates were obtained from soil collected at 5–10 cm below the surface in Nari Basin, Ulleung Island. Abalone and halibut samples were obtained from aquaculture farms in the corresponding sampling regions. The remaining marine organisms were obtained alive from local fishers in the corresponding regions and used immediately for bacterial isolation. Samples were transported to the laboratory under refrigerated conditions (4°C), and bacterial cultivation was initiated on the day of collection. Before cultivation, each sample was mixed with phosphate-buffered saline (PBS) at a 1:9 ratio and serially diluted up to 10^-2^. Subsequently, 100 μl of each dilution was spread onto MA (Kisan Bio, Korea) and incubated at 25°C for 24 h. After incubation, single colonies were visually selected based on colony color and size, and between 5 and 12 colonies were isolated from each sample. In total, 927 isolates were obtained and stored at −70°C as 20% glycerol stocks. Subsequently, the stock isolates were subcultured twice on MHA (Kisan Bio, Korea) to obtain single colonies. Only isolates that formed colonies after re-cultivation on MHA were selected for antimicrobial resistance testing using antibiotic disks.

### Taxonomic classification of bacterial isolates

Genomic DNA was extracted from the isolates using a Genomic DNA Extraction Kit (Bioneer, Korea). The bacterial 16S rRNA gene was then amplified by PCR using the extracted genomic DNA as a template and the universal primer set 27F (5′-AGAGTTTGATCMTGGCTCAG-3′) and 1492R (5′-TACGGYTACCTTGTTACGACTT-3′). PCR was performed with Solg^TM^ 2X Taq PCR Pre-Mix (SolGent, Korea) under the following cycling conditions: 30 cycles of denaturation at 95°C for 20 s, annealing at 55°C for 40 s, and extension at 72°C for 1 min. The PCR products were purified using a Genomic Gel Purification Kit (Bioneer, Korea), and the expected amplicon size of approximately 1.4 kb was confirmed by electrophoresis on a 1% agarose gel. Purified 16S rRNA gene amplicons were sequenced by Macrogen (Korea). Sequence reads of up to 700 bp were generated, and taxonomic identification was conducted using BLASTN against the NCBI database with a sequence similarity threshold of 97% or higher. To maintain conservative taxonomic resolution, isolates were assigned at the genus level, and species-level identification was not attempted.

### Antimicrobial susceptibility testing by the disk diffusion method

Antimicrobial susceptibility of the isolates recovered on MHA was evaluated by the disk diffusion method according to the CLSI M100 guidelines (CLSI, 2020). Before testing, each isolate was pre-cultured in MH broth at 35°C, and growth was monitored by measuring optical density at 600 nm (OD_600_) using an Epoch 2 Microplate Spectrophotometer (Agilent Technologies, USA).

Each isolate was inoculated onto MHA plates with a sterile cotton swab, and the plates were left for 5 min to allow absorption of the culture. Antibiotic disks were then placed on the agar surface. The six antibiotics used were selected based on non-human antimicrobial resistance information for livestock and fishery products provided by the One Health inter-ministerial antimicrobial resistance monitoring program (www.kdca.go.kr): chloramphenicol (CHL, 30 µg), trimethoprim/sulfamethoxazole (TMP/SMX, 25 µg), amoxicillin (AMX, 10 µg), streptomycin (STR, 25 µg), tetracycline (TET, 30 µg), and erythromycin (ERY, 15 µg). After incubation at 35°C for 18 h, inhibition zone diameters were measured using AntibiogramJ (Alonso et al., 2017). These antibiotics were selected to examine broad screening-level responses of isolates that were able to grow under standard MH conditions and included agents commonly used for comparing Gram-positive and Gram-negative bacterial responses. Inhibition-zone results were interpreted using CLSI M100 criteria only as practical reference cutoffs because taxon-specific CLSI breakpoints are not available for most environmental and marine-derived isolates examined in this study. Therefore, isolates below the corresponding reference cutoff were described as showing reduced susceptibility or putative resistance, rather than as clinically confirmed resistant isolates. *Enterococcus faecalis* ATCC 19433 (KCTC 3206) and *Escherichia coli* ATCC 11775 (KCTC 2441) were used as standard reference strains for Gram-positive and Gram-negative bacteria, respectively. Isolates showing reduced susceptibility to three or more antibiotics were classified as putative multidrug-resistant isolates.

### Determination of minimum inhibitory concentration

MIC testing was conducted using the broth dilution method under 2% NaCl conditions. Before MIC testing, the 562 isolates that showed limited or no detectable growth under standard MH conditions were screened for growth in MHB supplemented with 2% NaCl without antibiotics. Stable growth was defined as reproducible growth in antibiotic-free control wells across triplicate cultures, with a measurable increase in OD_600_ above the medium blank after incubation and without irregular growth among replicates. Isolates that failed to show reproducible growth in the control wells or produced inconsistent OD_600_ profiles were excluded from MIC analysis. Based on this screening, 115 isolates were selected for MIC testing.

Chloramphenicol, kanamycin, penicillin, tetracycline, and vancomycin were selected for MIC testing, and each antibiotic was prepared in a stepwise dilution series. For MIC testing, the antibiotics were selected based on their reproducible measurement under 2% NaCl-supplemented MHB and their representation of different antimicrobial classes under the modified broth condition. Prior to MIC determination, each bacterial isolate was pre-cultured in MH broth at 35°C. Standardized bacterial suspensions were then inoculated into liquid media containing each antibiotic and incubated at 35°C, and bacterial growth was evaluated based on OD_600_ measurements. To reduce analytical noise, OD_600_ values exceeding 1.0 were treated as 1.0, which was considered to represent the maximum growth plateau under the measurement conditions. The MIC was defined as the lowest antibiotic concentration that inhibited visible bacterial growth.

MIC measurements were performed under stepwise NaCl supplementation, and the results obtained at 35°C. *Escherichia coli* ATCC 25922 and *Enterococcus faecalis* ATCC 29212 were used as standard QC strains to confirm that the MIC assay was performed within a CLSI or European Committee on Antimicrobial Susceptibility Testing (EUCAST)-based quality control framework. In addition, MIC profiles of six reference strains were generated under the same 2% NaCl-supplemented MHB conditions and used as internal experimental reference ranges for comparison with marine-derived isolates. Because 2% NaCl supplementation is not a standard CLSI/EUCAST condition, these ranges were not used as official clinical breakpoints but only as practical reference ranges for interpreting MIC profiles under the modified medium condition used in this study and in the previous study (Han, 2026). Therefore, the disk diffusion results and MIC results were interpreted according to the growth condition and test format applied to each isolate group, rather than by direct comparison of all antibiotics between the two experiments.

### Statistical analysis

All experiments were performed in triplicate unless otherwise noted, and additional independent repeat experiments were conducted when necessary to confirm data consistency and reproducibility. Basal growth, inhibition zone diameter, and MIC-related measurements are presented as the mean and standard deviation based on repeated experiments.

## Results

### Taxonomic composition and distribution of bacterial isolates

In this study, a total of 927 bacterial isolates were recovered on MA from environmental samples, including lagoon water, seawater, and soil, as well as gut samples from various marine organisms, including abalone, crab, eel, halibut, and salmon. Of these, 505 isolates were obtained from environmental samples and 422 from gut samples. Analysis of the taxonomic composition of the isolates at the phylum level showed that the total isolate collection was dominated by *Proteobacteria* and *Firmicutes*. *Proteobacteria* accounted for the largest proportion, comprising 643 isolates, followed by *Firmicutes* with 228 isolates (Table 1). In most marine and brackish-water samples, *Proteobacteria* predominated; however, a distinct pattern was observed in the soil samples. Among the 39 isolates recovered from soil, 38 belonged to *Firmicutes*, indicating a taxonomic composition clearly distinct from that of the marine-associated samples. At the genus level, *Vibrio* represented the largest proportion of the total isolates, followed by *Pseudoalteromonas*, *Bacillus*, and *Shewanella* (Fig. 1A). *Vibrio* was detected in most environmental and gut samples except for soil and accounted for a relatively high proportion of isolates from seawater and marine organism gut samples (Fig. 1B).

The isolates recovered on MA were further examined for their ability to grow on MH medium, which is commonly used for standard antimicrobial susceptibility testing. Among the 927 total isolates, 365 showed growth on MH medium, whereas the remaining 562 showed no detectable growth under standard MH conditions. Comparison of growth on MH medium according to sample group showed that, in lagoon, seawater, and most marine organism-derived samples, a substantial proportion of isolates recovered on MA failed to grow on MH medium (Fig. S1). In contrast, all soil- and salmon-derived isolates grew on MH medium, indicating no apparent growth limitation under MH conditions in these samples. These results suggest that a considerable proportion of marine- and brackish-origin isolates may not grow sufficiently under standard MH conditions. Therefore, subsequent antimicrobial susceptibility analyses were performed separately according to growth capability on standard MH medium.

### Reduced susceptibility patterns among sample groups

Reduced susceptibility patterns were first examined by the disk diffusion method using the 365 isolates that were able to grow on MH medium (Table 2). Because taxon-specific interpretive criteria were not available for most isolates, the disk diffusion results were interpreted as screening-level reduced susceptibility based on practical CLSI reference cutoffs. The results indicated apparent differences in reduced susceptibility patterns among sample groups. Because the number of isolates differed among sample groups, the reduced susceptibility profiles in Table 2 should be interpreted as descriptive patterns rather than as statistically validated differences among sample groups. Among the environmental samples, lagoon-derived isolates frequently showed reduced susceptibility to trimethoprim/sulfamethoxazole, whereas seawater-derived isolates showed a relatively high frequency of reduced susceptibility to chloramphenicol. Soil-derived isolates exhibited high reduced susceptibility frequencies to chloramphenicol, trimethoprim/sulfamethoxazole, and amoxicillin, and all isolates recovered from soil showed reduced susceptibility to these antibiotics. Differences among sample groups were also observed among gut-derived isolates from marine organisms. Isolates from abalone, halibut, and salmon showed high reduced susceptibility frequencies to chloramphenicol and erythromycin, whereas isolates from crab and eel more frequently showed reduced susceptibility to trimethoprim/sulfamethoxazole and streptomycin.

Because *Vibrio* was the most dominant genus among the total isolates, multidrug resistance patterns of *Vibrio* isolates were further summarized separately (Table S1). Simultaneous reduced susceptibility to multiple antibiotics was frequently observed among *Vibrio* isolates, and the distribution of putative multidrug-resistant isolates differed among sample groups. Overall, the reduced susceptibility patterns observed in *Vibrio* were broadly similar to the major trends identified in the total isolates from the corresponding sample groups. In particular, most of the 32 *Vibrio* isolates recovered from abalone were resistant to chloramphenicol and erythromycin, and 30 of these isolates were classified as multidrug-resistant because they showed reduced susceptibility to three or more antibiotics. A high proportion of multidrug resistance was also observed among seawater-derived *Vibrio* isolates, with 7 of the 10 tested isolates classified as putative multidrug-resistant isolates. In addition, a small number of *Vibrio* isolates recovered from lagoon, halibut gut, and salmon gut samples showed reduced susceptibility patterns similar to those observed in the total isolate collections from the respective sample groups. In contrast, no multidrug resistance was detected among *Vibrio* isolates recovered from eel.

### MIC patterns under MHB supplemented with 2% NaCl

Among the 562 isolates that showed no detectable growth under standard MH conditions, MIC analysis was performed for 115 isolates that showed reproducible growth in antibiotic-free MHB supplemented with 2% NaCl across triplicate cultures (Table 3). These isolates comprised diverse taxonomic groups, among which *Vibrio* accounted for the largest proportion with 50 isolates. MIC analysis was conducted to quantitatively evaluate growth inhibition patterns across antibiotic concentrations (Fig. 2). The MIC patterns differed clearly depending on the antibiotic tested. Chloramphenicol and tetracycline inhibited the growth of most isolates at relatively low concentrations. In particular, tetracycline showed a marked inhibitory effect at 1 μg/ml, whereas chloramphenicol showed clear growth inhibition at 8 μg/ml. Compared with the internally generated MIC ranges of the six reference strains analyzed under the same 2% NaCl conditions, these distributions indicate relatively low MIC values for chloramphenicol and tetracycline.

For kanamycin, a concentration-dependent decrease in growth was observed within the range of 32–64 μg/ml, which was similar to the MIC range observed for the standard reference strains *Bacillus clausii* and *Bacillus cereus*. For vancomycin, a reduction in growth was observed at 1,024 μg/ml, showing a pattern similar to the MIC range observed for the standard Gram-negative reference strains *Salmonella enterica* and *Escherichia coli*. In contrast, penicillin showed only limited inhibitory effects in many of the isolates. Some isolates derived from lagoon and crab samples exhibited growth inhibition within a range similar to the MIC reference range of the standard strains, whereas isolates from seawater, abalone, and eel showed only limited growth inhibition within the tested concentration range (Fig. 2). Meanwhile, the halibut-derived isolates were represented by only a single *Vibrio* isolate, which limited interpretation of sample group-specific MIC patterns.

Similar to the overall pattern observed across the total isolates, *Vibrio* isolates showed low MIC values for tetracycline and chloramphenicol, whereas growth was maintained at high concentrations of penicillin and vancomycin (Fig. 3). For most antibiotics except penicillin, the MIC distributions of *Vibrio* isolates were comparable to the internally generated MIC ranges of the Gram-negative reference strains *Salmonella enterica* and *Escherichia coli* under the same 2% NaCl-supplemented MHB conditions.

## Discussion

In this study, we evaluated the applicability and limitations of standard antimicrobial susceptibility testing conditions using bacterial isolates recovered from lagoon, seawater, soil, and marine organism gut samples. Among the 927 total isolates, only 365 isolates grew on standard MH medium, whereas 562 isolates were not evaluable under standard MH conditions. These results indicate that a substantial proportion of marine- and brackish-origin bacteria may not be adequately evaluated under conventional antimicrobial susceptibility testing conditions. In particular, the frequent growth limitation observed in lagoon, seawater, and most marine organism-derived isolates suggests that the physiological growth requirements of these bacteria should be considered before susceptibility testing is performed. For gut-derived isolates, sample origin and incomplete background information should be considered as additional factors affecting interpretation. Because detailed rearing or capture histories were not available for all marine organism specimens, previous antimicrobial exposure or other background factors cannot be excluded. These factors may have influenced the gut bacterial communities and reduced susceptibility profiles and should therefore be considered potential confounding variables.

The disk diffusion assay using the 365 isolates that were able to grow on standard MH medium showed that reduced susceptibility patterns differed among sample groups. These observed reduced susceptibility profiles should be interpreted cautiously, particularly because the number of isolates differed among sample groups. Lagoon-, seawater-, and soil-derived isolates each showed relatively high reduced susceptibility frequencies to different antibiotics, and similar differences among sample groups were also observed among isolates from abalone, crab, eel, halibut, and salmon guts. These findings suggest that the culturable bacterial populations recovered from different sample groups had distinct phenotypic profiles of reduced susceptibility. However, because the bacterial composition varied among sample groups, the observed differences should not be attributed solely to a specific contamination source or antibiotic selection pressure. Instead, they should be interpreted in the context of the taxonomic composition of the culturable isolates recovered from each sample group.

From this perspective, the distribution and reduced susceptibility profiles of *Vibrio* were examined separately. In this study, *Vibrio* was the most dominant genus and was detected in most environmental and marine organism gut samples except for soil. Previous studies have also shown that *Vibrio* is widely distributed in marine and brackish environments and can reflect ecological differences among coastal habitats as well as variation in antimicrobial resistance phenotypes (Di et al., 2019; Hobe et al., 2026). The *Vibrio* isolates in this study also showed differences in reduced susceptibility patterns among sample groups. In particular, 30 of the 32 *Vibrio* isolates recovered from abalone showed reduced susceptibility to three or more antibiotics and were therefore classified as putative multidrug-resistant isolates. A similarly high proportion of putative multidrug resistance was observed among seawater-derived *Vibrio* isolates, with 7 of 10 classified as putative multidrug-resistant isolates. In addition, the reduced susceptibility patterns observed in *Vibrio* were generally similar to the major trends identified in the total isolate collections from the corresponding sample groups. These results suggest that *Vibrio* may be considered a potential candidate taxonomic group for further evaluating reduced susceptibility patterns in marine-derived bacteria, rather than a definitive indicator taxon.

For the 562 isolates that were not evaluable under standard MH conditions, MIC analysis was performed under MHB supplemented with 2% NaCl. This analysis was intended to quantitatively examine antibiotic responses in marine-derived isolates that could not be evaluated under standard susceptibility testing conditions. Among these isolates, 115 showed stable growth under 2% NaCl-supplemented conditions and were therefore subjected to MIC analysis. MIC patterns varied according to antibiotic type. Chloramphenicol and tetracycline inhibited the growth of most isolates at relatively low concentrations, kanamycin showed a concentration-dependent decrease in growth, and vancomycin inhibited growth only at 1,024 μg/ml, consistent with the internally generated MIC range observed for the Gram-negative reference strains *Salmonella enterica* and *Escherichia coli* under the same 2% NaCl-supplemented MHB conditions. In contrast, reduced susceptibility to penicillin was relatively high in isolates from seawater, abalone, and eel.

These MIC ranges should therefore be interpreted as internal experimental reference ranges under the modified 2% NaCl-supplemented condition, rather than as official CLSI/EUCAST clinical breakpoints. When the MIC distributions of *Vibrio* isolates were examined separately, they generally reflected the overall antibiotic response patterns observed in the total isolates. For most antibiotics except penicillin, the MIC distributions of *Vibrio* were also comparable to the internally generated MIC ranges of the Gram-negative reference strains *Salmonella enterica* and *Escherichia coli* under the same 2% NaCl-supplemented MHB conditions. If the relatively high reduced susceptibility to penicillin observed in *Vibrio* reflects the resistance characteristics of the seawater-, abalone-, and eel-derived isolates, *Vibrio* may be considered a potential candidate taxonomic group for evaluating NaCl-adjusted antimicrobial susceptibility patterns in marine-derived bacteria. However, this interpretation should be considered preliminary and should be confirmed using higher-resolution molecular or genomic approaches because taxonomic identification was based on partial 16S rRNA gene sequences of approximately 700 bp. In addition, the present study did not include genetic or genomic analyses of antimicrobial resistance determinants. Therefore, the reduced susceptibility and putative multidrug resistance observed in disk diffusion and MIC assays should be interpreted as phenotypic profiles, and their underlying genetic basis requires further investigation using targeted resistance-gene screening or whole-genome sequencing.

Although both the disk diffusion assay and MIC analysis were used in this study, the two tests were applied to different isolate groups and were not designed for direct comparison across all antibiotics because the antibiotics used in the two assays were not completely identical. The disk diffusion assay was performed with isolates that were able to grow on standard MH medium, whereas MIC analysis was performed with isolates that were not evaluable under standard MH conditions but showed stable growth in 2% NaCl-supplemented MHB. Accordingly, the two datasets were interpreted primarily within each test condition. Therefore, even when the same antibiotics (chloramphenicol and tetracycline) were included in both tests, the results should not be interpreted simply as concordant or discordant because the tested isolate groups, medium conditions, and readout criteria were different. Instead, these two antibiotics provided only limited shared points of comparison between the disk diffusion and MIC results. Thus, differences between the disk diffusion and MIC results may reflect not only differences in isolate characteristics, but also differences in test format, growth medium, and endpoint measurement. This difference is illustrated by the chloramphenicol results. In the disk diffusion assay, relatively high chloramphenicol reduced susceptibility was observed in isolates from some sample groups. In contrast, MIC analysis under 2% NaCl-supplemented MHB showed that chloramphenicol inhibited the growth of most tested isolates at relatively low concentrations. Rather than indicating a contradiction, this result suggests that antibiotic response patterns can differ depending on the isolate group and test condition. Thus, results obtained under standard MH conditions alone are unlikely to represent the responses of the entire isolate collection, and both pre-test growth capability and medium condition should be considered in antimicrobial susceptibility evaluation of marine-derived bacteria. Future studies should include representative isolates tested by both disk diffusion and MIC under matched antibiotic and medium conditions to further evaluate the relationship between the two assay formats.

Taken together, our results suggest that standard MH conditions alone may be insufficient to evaluate antibiotic responses in a substantial proportion of marine- and brackish-origin isolates. This study extends the previous reference-strain-based evaluation (Han, 2026) to diverse isolates from coastal environments and marine organism guts by separating isolates according to their growth capability under standard MH conditions and applying 2% NaCl-supplemented MHB as a supplementary condition to isolates excluded from standard testing. Although *Vibrio* is widely recognized as a dominant bacterial group in marine environments, existing studies have largely focused on specific pathogenic species such as *Vibrio cholerae* and *Vibrio parahaemolyticus* (Di et al., 2019; Hobe et al., 2026; Park et al., 2018). By contrast, efforts to evaluate *Vibrio* as a broader reference taxon for antimicrobial susceptibility assessment have been limited. In this study, *Vibrio* was repeatedly recovered from multiple sample groups and showed antibiotic-dependent response patterns. These findings indicate that further studies on *Vibrio* may help refine NaCl-adjusted antimicrobial susceptibility testing conditions for marine-derived bacteria and evaluate whether selected marine-associated taxa can provide practical reference information for future susceptibility assessment.

## Figures and Tables

**Fig. 1.**
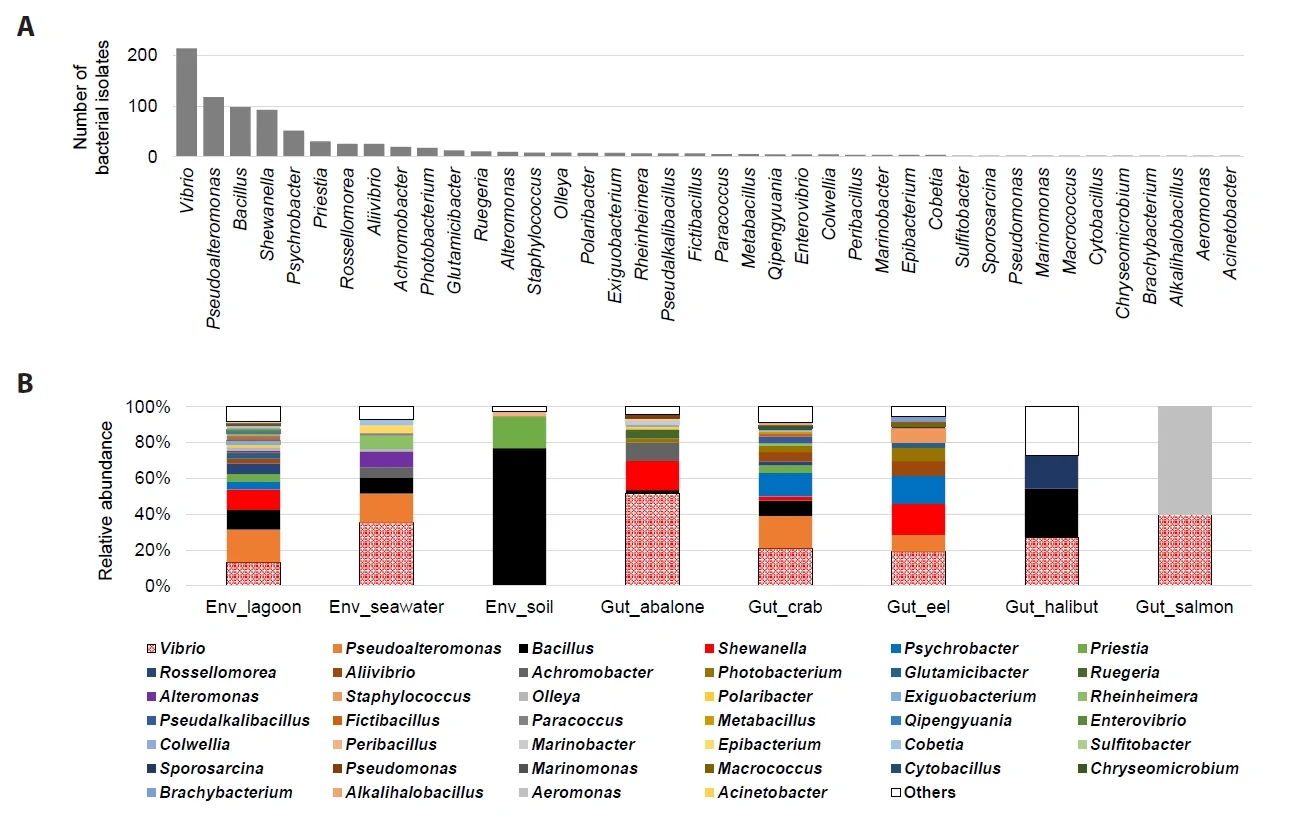
Composition and distribution of bacterial isolates from environmental and gut samples. (A) Genus-level distribution of total bacterial isolates, excluding genera represented by two or fewer isolates. (B) Group-specific relative abundance of bacterial isolates. Taxa with an overall relative abundance of < 1% across samples were grouped as Others.

**Fig. 2.**
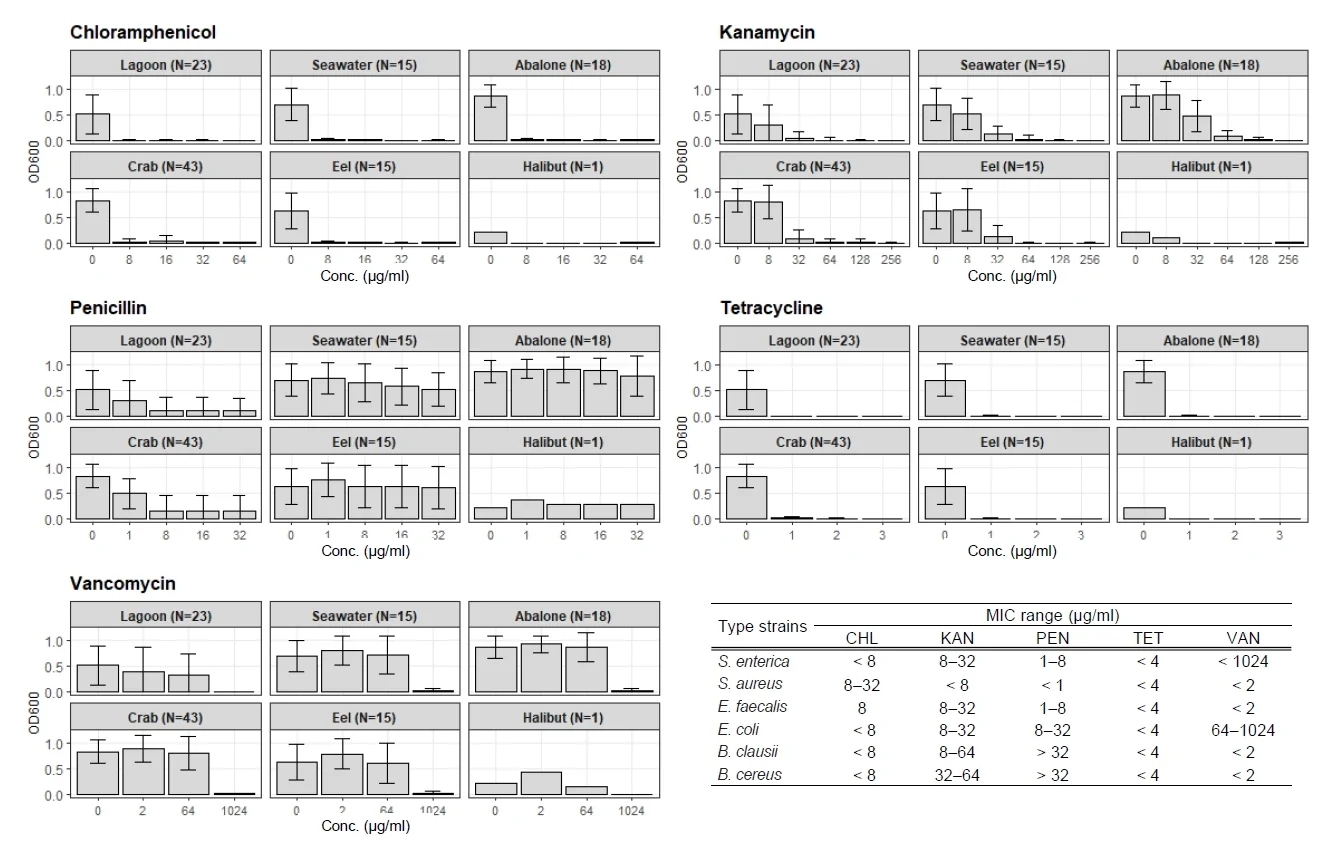
MIC profiles of bacterial isolates recovered from environmental and marine organism gut samples in 2% NaCl-supplemented MHB. MIC profiles for six reference strains were generated under the same 2% NaCl-supplemented MHB conditions, as shown in Fig. S2. The corresponding internally generated MIC reference ranges are shown in the lower right panel and were not used as official CLSI/EUCAST clinical breakpoints.

**Fig. 3.**
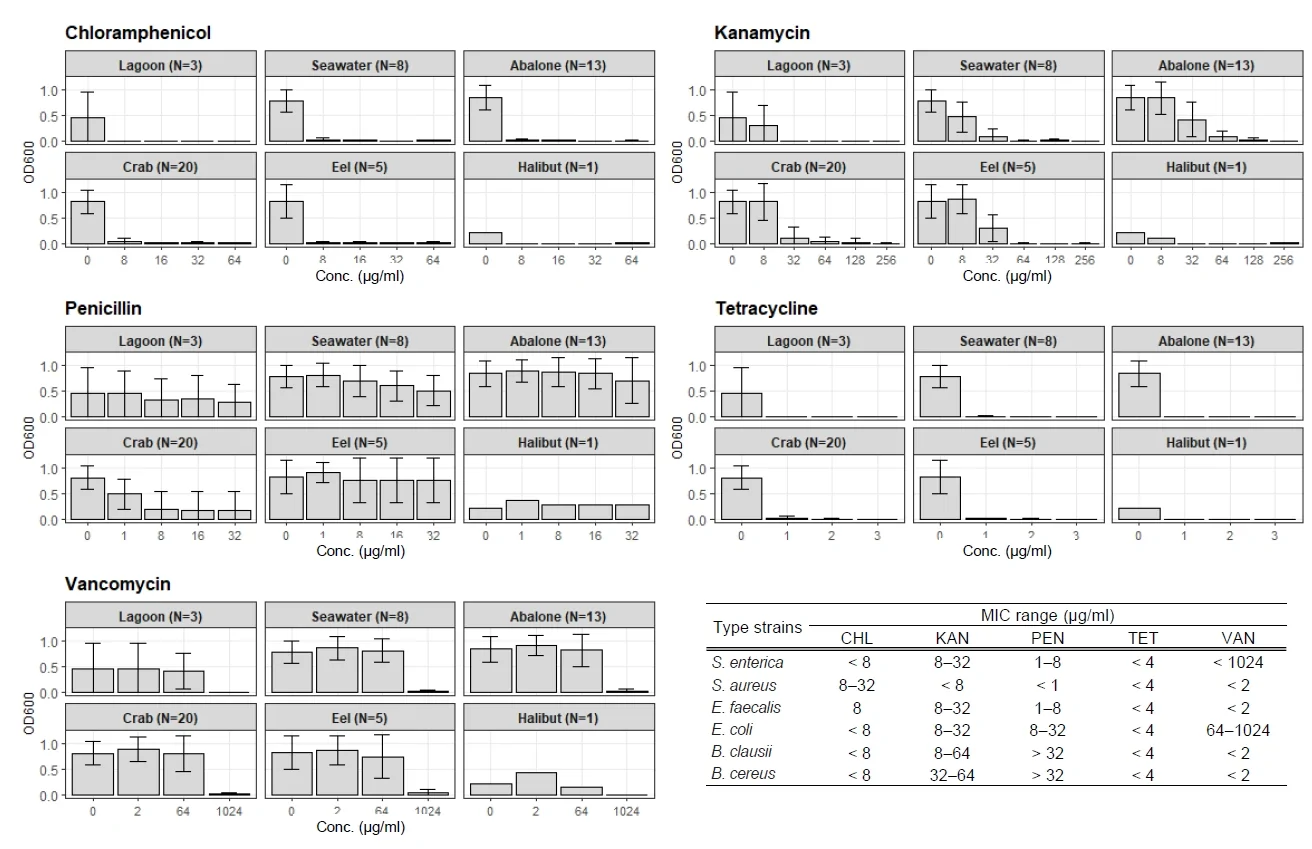
MIC profiles of *Vibrio* isolates recovered from environmental and marine organism gut samples in 2% NaCl-supplemented MHB. MIC profiles for six reference strains were generated under the same 2% NaCl-supplemented MHB conditions, as shown in Fig. S2. The corresponding internally generated MIC reference ranges are shown in the lower right panel and were not used as official CLSI/EUCAST clinical breakpoints.

**Table 1.** Taxonomic composition of bacterial isolates recovered from environmental and gut samples

Number of bacterial isolates from				Taxonomy				
Group		Location	Date (Year/Month)	Proteobacteria	Firmicutes	Actinobacteria	Bacteroidota	Aquificota
Environment (N = 505)	Lagoon (N = 398)	Gangneung	2022/05–2023/06	244	121	15	18	0
	Seawater (N = 68)	Ulleung	2021/07	60	6	0	2	0
	Soil (N = 39)	Ulleung	2021/07	1	38	0	0	0
Gut (N = 422)	Abalone (N = 159)	Wando	2021/07	151	3	3	2	0
	Crab (N = 138)	Uljin	2022/08	93	40	4	1	0
	Eel (N = 109)	Yeosu	2022/06	85	13	10	0	1
	Halibut (N = 11)	Busan	2021/07	4	7	0	0	0
	Salmon (N = 5)	Yangyang	2021/07	5	0	0	0	0

**Table 2.** Reduced susceptibility profiles of bacterial isolates recovered on MH according to sample group

Group (N = 365)	Num. of isolates with reduced susceptibility to					
	CHL	TMP/SMX	AMX	STR	TET	ERY
Lagoon (N = 119)	75 (63%)	105 (88%)	75 (63%)	55 (46%)	28 (24%)	29 (24%)
Seawater (N = 32)	32 (100%)	18 (56%)	1 (3%)	11 (34%)	17 (53%)	20 (63%)
Soil (N = 39)	39 (100%)	39 (100%)	39 (100%)	0 (0%)	0 (0%)	9 (23%)
Abalone (N = 81)	81 (100%)	74 (91%)	49 (60%)	17 (21%)	29 (36%)	74 (91%)
Crab (N = 30)	0 (0%)	25 (83%)	11 (37%)	23 (77%)	0 (0%)	1 (3%)
Eel (N = 50)	3 (6%)	17 (34%)	11 (22%)	17 (34%)	3 (6%)	5 (10%)
Halibut (N = 9)	9 (100%)	6 (67%)	0 (0%)	1 (11%)	0 (0%)	3 (33%)
Salmon (N = 5)	5 (100%)	5 (100%)	0 (0%)	1 (20%)	0 (0%)	5 (100%)

**Table 3.** Genus-level distribution of bacterial isolates (N = 115) grown in MHB under 2% NaCl conditions across environmental and marine organism gut samples

Num. of taxa (Genus)		Lagoon (N = 23)	Seawater (N = 15)	Abalone (N = 18)	Crab (N = 43)	Eel (N = 15)	Halibut (N = 1)
*Vibrio*	50	3	8	13	20	5	1
*Pseudoalteromonas*	15	3	3	0	6	3	0
*Photobacterium*	9	0	0	2	1	6	0
*Shewanella*	6	2	0	2	2	0	0
*Rossellomorea*	6	6	0	0	0	0	0
*Chryseomicrobium*	3	0	0	0	3	0	0
*Exiguobacterium*	3	3	0	0	0	0	0
*Epibacterium*	2	0	2	0	0	0	0
*Aliivibrio*	2	0	0	0	2	0	0
*Psychrobacter*	2	0	0	0	2	0	0
*Catenococcus*	2	2	0	0	0	0	0
*Glutamicibacter*	2	2	0	0	0	0	0
*Bacillus*	1	0	1	0	0	0	0
*Alteromonas*	1	0	1	0	0	0	0
*Ruegeria*	1	0	0	1	0	0	0
*Oceanihabitans*	1	0	0	0	0	1	0
*Alkalihalobacillus*	1	1	0	0	0	0	0
*Kytococcus*	1	0	0	0	1	0	0
*Lysinibacillus*	1	0	0	0	1	0	0
*Rhodococcus*	1	0	0	0	1	0	0
*Pseudalkalibacillus*	1	0	0	0	1	0	0
*Cognaticolwellia*	1	0	0	0	1	0	0
*Solibacillus*	1	0	0	0	1	0	0
*Rheinheimera*	1	0	0	0	1	0	0
*Jeotgalibacillus*	1	1	0	0	0	0	0

## Supplementary Information

Table S1. Antibiotic resistance profiles of Vibrio isolates recovered on MH according to sample source.

Fig. S1. Distribution of bacterial isolates recovered on MH and non-MH (MA) media according to sample source.

Fig. S2. MIC profiles of quality control strains under 2% NaCl conditions.
