# Supplementary material for: Application of NaCl-supplemented antimicrobial susceptibility testing conditions to marine bacterial isolates
Source: J Microbiol. 2026 Sep 30;64(9):e2606005. doi: 10.71150/jm.2606005 (PMC13714652; doi:10.71150/jm.2606005)
Supplement: Supplementary file 1 [file jm-2606005-Supplementary-Fig-S1.pdf]

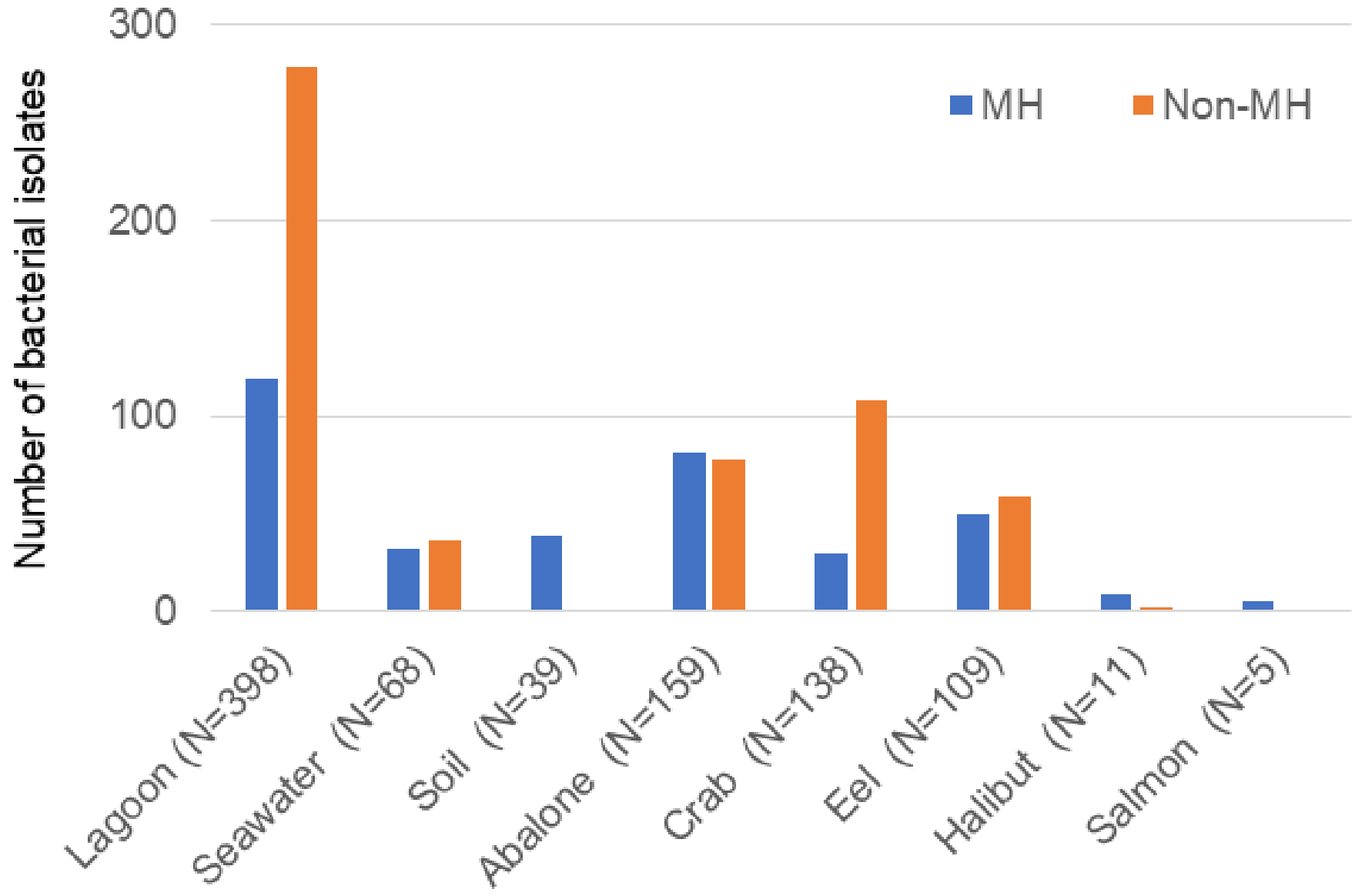

Fig. S1. Distribution of bacterial isolates recovered on MH and non-MH (MA) media according to sample source.
