# Supplementary material for: Application of NaCl-supplemented antimicrobial susceptibility testing conditions to marine bacterial isolates
Source: J Microbiol. 2026 Sep 30;64(9):e2606005. doi: 10.71150/jm.2606005 (PMC13714652; doi:10.71150/jm.2606005)
Supplement: Supplementary file 2 [file jm-2606005-Supplementary-Fig-S2.pdf]

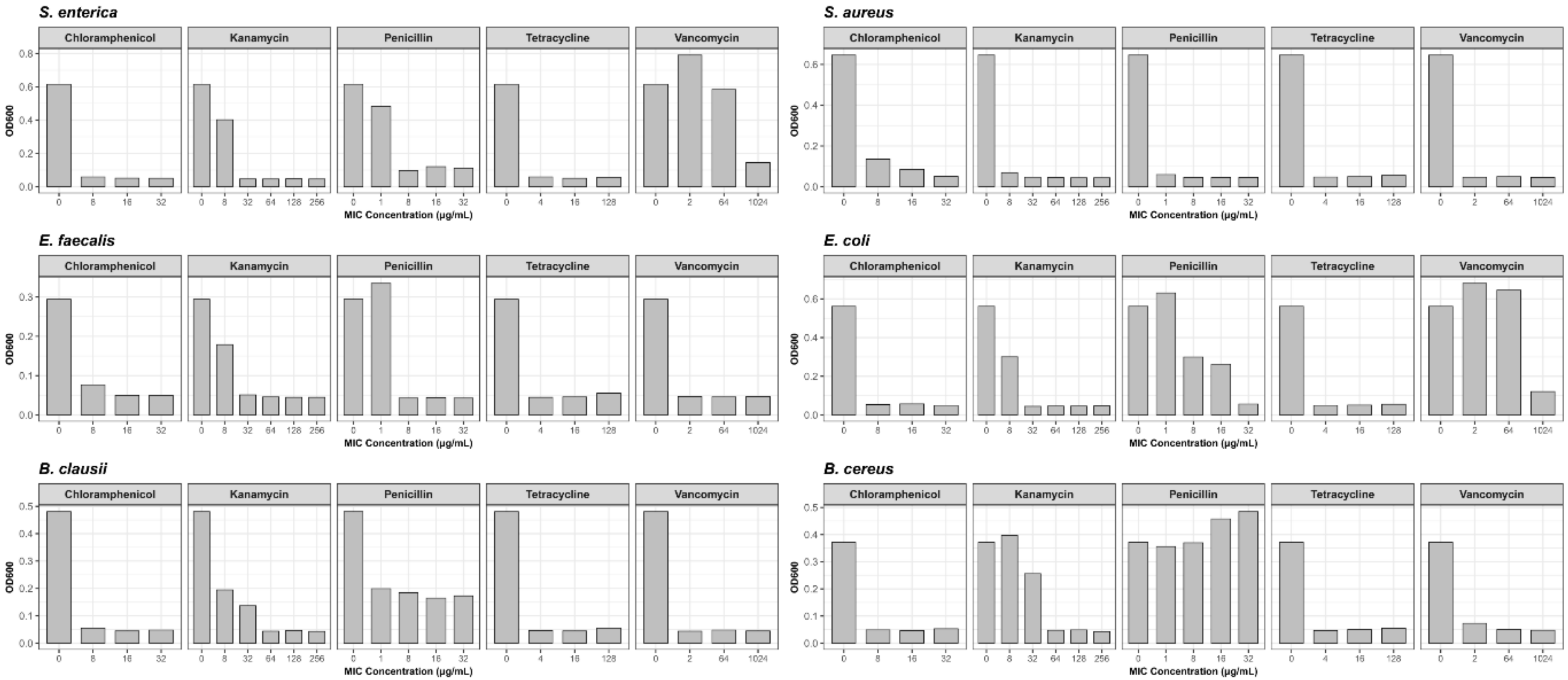

Fig. S2. MIC profiles of quality control strains under 2% NaCl conditions.
