# Supplementary material for: Application of NaCl-supplemented antimicrobial susceptibility testing conditions to marine bacterial isolates
Source: J Microbiol. 2026 Sep 30;64(9):e2606005. doi: 10.71150/jm.2606005 (PMC13714652; doi:10.71150/jm.2606005)
Supplement: Supplementary file 3 [file jm-2606005-Supplementary-Table-S1.pdf]

Table S1. Antibiotic resistance profiles of *Vibrio* isolates recovered on MH according to sample source

Source	Resistant *Vibrio* isolates to						
	CHL	TRI/SUL	AMX	STR	TET	ERY	MDR
Lagoon (N = 5)	3	3	2	0	0	1	2
Seawater (N = 10)	10	7	1	5	6	8	7
Soil (N = 0)	0	0	0	0	0	0	0
Abalone (N = 32)	32	27	23	2	6	31	30
Crab (N = 0)	0	0	0	0	0	0	0
Eel (N = 12)	0	0	4	0	0	0	0
Halibut (N = 2)	2	2	0	0	0	0	0
Salmon (N = 2)	2	2	0	1	0	2	2
